# Metabolic and inflammatory linkage of the chicken cecal microbiome to growth performance

**DOI:** 10.3389/fmicb.2023.1060458

**Published:** 2023-02-23

**Authors:** Liqi Wang, Fuping Zhang, Hui Li, Shenglin Yang, Xiang Chen, Shuihua Long, Shenghong Yang, Yongxian Yang, Zhong Wang

**Affiliations:** ^1^Key Laboratory of Animal Genetics, Breeding and Reproduction in the Plateau Mountainous Region, Ministry of Education, Guizhou University, Guiyang, Guizhou, China; ^2^College of Animal Sciences, Guizhou University, Guiyang, Guizhou, China; ^3^School of Public Health, Xinyu University, Xinyu, Jiangxi, China

**Keywords:** indigenous chickens, market-weight, cecal microbiota, serum metabolome, inflammatory cytokine

## Abstract

**Introduction:**

Chinese indigenous chicken breeds are widely used as food in China but their slow growth rate and long farming cycle has limited their industrial production.

**Methods:**

In the current study we examined whether the market weights of native chicken breeds were related to specific cecal bacteria, serum metabolites and inflammatory cytokines. We examined cecal bacterial taxa using 16S rDNA analysis along with untargeted serum metabolites and serum inflammatory cytokines.

**Results:**

We found that the cecal microbiota could explain 10.1% of the individual differences in chicken weights and identified key cecal bacterial genera that influenced this phenotype. The presence of *Sphaerochaeta* spp. improved growth performance via bovinic acid metabolism. In contrast, *Synergistes* and *norank_f_Desulfovibrionaceae* had a negative effect on growth by inducing expression of the inflammatory cytokine IL-6.

**Discussion:**

We were able to link specific bacterial genera with growth promotion in chickens and this study will allow further development of their use as probiotics in these animals.

## Introduction

Growth performance is an important economic trait for broiler chickens and antibiotics supplied in feed have been traditionally used to increase the market weight of these animals. However, these types of intensive antibiotic-use practices have accelerated the development of antibiotic resistance in bacteria and this has become a major public global health concern (Wang et al., 2019). Therefore, many countries including China have prohibited the use of antibiotics as growth promoters in food animal production and scientists are committed to developing antibiotic alternatives for animal growth promotion (Zhang et al., 2022a). One promising alternative is the use of probiotics or associated metabolites derived from the chicken gut microbiome (Ayalew et al., 2022). The gut microbiota that are present in animal gastrointestinal tracts form a diverse, complex and dynamic ecosystem composed of tens of millions of microorganisms (Glendinning et al., 2019). These microorganisms play essential roles in maintaining animal health and their primary site of residence in birds is the cecum (Cui et al., 2021; Zhang et al., 2022b).

There is direct evidence that the cecal microflora of chickens has a positive impact on growth performance. For example, sex differences in chicken growth performance were related to glycan and lipid metabolic functions of cecal bacteria (Cui et al., 2021). Another study demonstrated that transplantation of fecal microbiota and prebiotic supplementation promoted daily weight gains for chickens (Li et al., 2016). In particular, the use of the probiotic *Lactobacillus plantarum P-8* in broiler diets increased adsorption of recalcitrant polysaccharides that were converted to a nutrition source and thus improved feed efficiency (Borda-Molina et al., 2018). Specific bacteria have also been linked to chicken growth traits and these include *Microbacterium* spp. and *Sphingomonas* spp. in Turpan × White Leghorn hybrids that were beneficial to chicken growth while the presence of *Slackia* spp. promoted a growth-inhibiting effect on chickens (Zhang et al., 2022b). Studies such as these have indicated that gut microbiota is a potential target for regulating growth performance.

Despite established relationships between gut the microbiota and chicken growth performance, mechanistic details have yet to be formulated. However, intestinal inflammation due to colonization by bacterial pathogens has been linked to poor growth performance and probiotic treatments can counter these effects (Zhang et al., 2022a). Regulation of fat metabolism and improved growth performance have also been directly linked to specific cecal microbiota (Zhang et al., 2022b). However, more precise mechanistic details are lacking because metabolic functional pathway assignments for the key microbiota were based on 16S rDNA and metagenomic sequencing that predict, but do not prove, functional relationships (Wen et al., 2019; Elokil et al., 2020; Darwish et al., 2021; Wen et al., 2021). In contrast, metabolomics have been used to fill the information gap between gene and phenotype although metabolomics has not been directly applied to chicken growth performance (Wu et al., 2018; Liu et al., 2021). Therefore, in the current study, we integrated and interpreted metabolomic information to construct a metabolic network of chicken growth traits to gain a new perspective and identify underlying biological processes. We hypothesized that specific cecal microorganisms in chickens can regulate host serum metabolites as well as inflammatory cytokine production and thereby affect market weight. We used Qiandongnan Xiaoxiang chickens, Guizhou yellow broilers and Wumeng black-bone chickens as examples of the most common indigenous chickens used for food production in Guizhou Province, China (Xu, 2018). Our goal was to link key cecal bacterial taxa to chicken market weight and integrate this with serum metabolomic and serum cytokine analyses to identify phenotypic regulatory mechanisms. Our results can be used to guide design of microbial or probiotic intervention targets that regulate market weight. These will help establish new options for the development of antibiotic alternatives.

## Materials and methods

### Animals and sample collection

Two cohorts of indigenous chickens in Guizhou Province, China were collected in this study and the experimental cohort consisted of 60 Qiandongnan Xiaoxiang chickens that were raised to 160 ± 3 days old and 12 with the highest (1.78 ± 0.12 kg, 6 of each sex) and the lowest (1.02 ± 0.10 kg, 6 of each sex) body-weights were selected for further study. In the validation trail, 79 chickens including 55 Guizhou yellow broilers (1.89 ± 0.28 kg, *n* = 55, 26 males and 29 females) and 24 Wumeng black-bone chickens (1.61 ± 0.22 kg, 11 males and 13 females) were collected as validation cohorts. All chickens in the same cohort were raised in the same chicken house and were provided identical commercial diets with *ad libitum* access to water and feed and were managed in the same way. Chickens were slaughtered at the age of 160 ± 3d and antibiotics were not used for 1 month prior to sample collection. We collected 24 each of blood and cecal content samples from the experimental cohort and 79 each from the validation cohort (Supplementary Table S1). Serum was collected from blood following centrifugation using standard procedures. Since serum was failed to be obtained from blood samples of three Qiandongnan Chickens, 21 serum samples (high-market-weight group, *n* = 12; low-market-weight group, *n* = 9 ) were involved for metabolomics analysis. Cecal content samples were collected at the same position of the intestinal tract and immediately frozen in liquid nitrogen for transport to the laboratory and then stored at −80°C.

Serum and cecal content samples from the experimental cohorts were employed for metabonomic and microbiota analysis using ultra-high-performance liquid chromatography—tandem mass spectrometry (UPLC-MS/MS) and 16S rDNA gene sequencing, respectively. Serum and cecal content samples from the validation cohort were used for cytokine determinations and microbiota analysis by enzyme-linked immunosorbent assay (ELISA) and 16S rDNA gene sequencing, respectively.

### DNA manipulations and sequencing

Total microbial DNA were extracted from 103 cecal content samples using the Magnetic Soil and Stool DNA Kit (Tiangen, Beijing, China) according to the manufacturer’s protocol. DNA concentrations and purity were determined by UV spectroscopy using a NanoDrop*-*1000 instrument (Thermo Fisher, Pittsburg, PA, United States) and 0.8% agarose gel electrophoresis. The V3–V4 region of the bacterial 16S rRNA gene was amplified with the primer pair 338F(5′-ACTCCTACGGGAGGCAGCA-3′)and 806R(5′-GGACTACHVGG GTWTCTAAT-3′) that were combined with adapter and barcode sequences. PCR amplicons were quantified using Quant-iT dsDNA HS reagent (Thermo Fisher) and pooled.

High-throughput sequencing analysis of bacterial rDNA genes in the purified and pooled samples were performed on an Illumina Hiseq 2500 platform (Illumina, San Diego, CA, United States). Trimmomatic software (version 0.33; Bolger et al., 2014) was used to filter out primers, low-quality and ambiguous sequences. Cut adapt (version 1.9.1; Lindgreen, 2012) was used to identify and remove primer sequences. The resulting paired-end reads from the clean data sets were assembled into tags using FLASH (version 1.2.11; Magoc and Salzberg, 2011). The sequence depth of each sample was rarefied to 75,007 tags to avoid statistical bias resulting from an uneven sequencing depth. USEARCH (version 10.0; Edgar, 2013) was employed to cluster tags of >97% identity into operational taxonomic units (OTU) and the OTU filtering threshold was set at 0.005% (Bokulich et al., 2013). RDP classifier (version 2.2; Wang et al., 2007) was used to produce OTU taxonomic assignments and representative sequences of each OTU were compared using the Silva reference database (Release 132, http://www.arb-silva.de; Quast et al., 2013) for OTU annotations.

### Determination of serum metabolomic profiles in Qiandongnan Xiaoxiang chickens

An untargeted metabolomic analysis of serum samples from the Qiandongnan Xiaoxiang chicken were conducted by a commercial company (Shanghai Biotree Biotech, Shanghai, China). The analyses were performed using UPLC-MS/MS as previously described (Dunn et al., 2011; Cai et al., 2015; Wang et al., 2016). In brief, a Vanquish high-performance liquid chromatography (HPLC) system (Thermo Fisher) equipped with a BEH amide column (2.1 mm × 100 mm, 1.7 μm) coupled to an Orbitrap MS Q Exactive HFX mass spectrometer (Thermo Fisher) was used for separations. The mobile phase consisted of (Solvent A) 25 mM ammonium acetate and 25 mM ammonia hydroxide in water pH = 9.75 and (Solvent B) acetonitrile. The auto-sampler temperature was 4°C and the injection volume was 2 μL. The QE HFX mass spectrometer was used for its ability to acquire MS/MS spectra on the information-dependent acquisition (IDA) mode and was controlled by the Xcalibur acquisition software supplied with the instrument. In this mode, the full scan MS spectrum was continuously evaluated. The ESI source conditions were set as follows: sheath gas flow rate, 30 Arb; Aux gas flow rate, 25 Arb; capillary temperature, 350°C; full MS resolution, 60,000; MS/MS resolution, 7,500; collision energy, 10/30/60 in NCE mode, spray voltage, 3.6 kV (positive) or −3.2 kV (negative).

The raw data were converted to the extensible markup language (mzXML) format using ProteoWizard and processed with an in-house program developed using R language and based on XCMS for peak detection, extraction, alignment and integration (Smith et al., 2006). An in-house MS_2_ database (BiotreeDB) was used for metabolite annotations with a set cutoff of 0.3. Metabolites were identified using the HMDB database^1^ and endogenous metabolites were reserved for further construction of metabolite feature modules. The internal standard normalization method was employed in this data analysis. The final dataset containing peak numbers, sample names and normalized peak areas were imported into SIMCA 16.0.2 (Sartorius Stedim Data Analytics AB, Umea, Sweden) for multivariate analysis (Wiklund et al., 2008).

### Serum cytokine measurements from Guizhou yellow broilers and Wumeng black-bone chickens

Commercial ELISA kits (Ziker Biological Technology, Shenzhen, China) were used to quantify levels of INF-*γ*, IL-1*β*, IL-5, IL-6, IL-17, and IL-22 in the sera of the 79 chickens in the validation cohorts according to the manufacturer’s protocol. Detection limits were 5 pg./mL (IFN-γ), 40 pg./mL (IL-1*β*), 5 pg./mL (IL-5), 2 pg./mL (IL-6), 3 pg./mL (IL-17), and 2 pg./mL (IL-22).

### Statistical analyses

#### Calculation and comparation of α- and β-diversity of cecal microbiota

OTUs with relative abundances >0.01% that were present in >10% of individuals were used for further analysis. Mothur software (version 1.31.2; Schloss et al., 2009) was employed to calculate the α-diversity of OTUs including Shannon, Simpson, Chao1, Faith’s phylogenetic diversity (PD) and ACE indices (Shannon, 1948; Chao, 1984; Faith, 1992). *β-*diversity of chicken cecal microbial community between high-and low-market-weight chicken groups was calculated using principal coordinate analysis (PCoA) based on unweighted UniFrac distances using QIIME (Caporaso et al., 2010).

#### Construction of microbial co-abundance groups

OTUs whose relative abundance was >0.05% and were present in >20% of all samples were selected for co-abundance group (CAG) construction. SPIEC-EASI package in R was employed to cluster CAGs. Interactions between OTUs were calculated based on their abundances using the SparCC algorithm with 100 bootstrap replicates followed by computing correlation matrices (Friedman and Alm, 2012). The Spearman’s rank correlation coefficients of pairwise OTUs >0.55 were used to classify CAGs. The correlation coefficient values were converted to a correlation distance (1-correlation coefficient value) and the OTUs were clustered into CAGs using the Ward clustering algorithm with a ‘hclust’ function in the SPIEC-EASI R package. Permutational MANOVA was applied to detect the statistical significance of each CAG clustering using 999 permutations with Bray–Curtis dissimilarity. The CAG was deemed acceptable when *p <* 0.005. Wilcoxon rank sum tests were performed to identify differences of relative abundance at the CAG level between the high-market-weight chickens and low-market-weight chickens. The CAG network was visualized in Cytoscape V. 3.7.1 (Lopes et al., 2010).

#### Identification of cecal microbiota associated with market-weight in the validation cohorts using two-part model

A two-part model was used to identify OTUs which that were linked with chicken market-weight in the validation trail (Fu et al., 2015). Specifically, a binomial analysis was performed to determine associations between the presence or absence of an OTU and market-weight. For a particular OTU, each sample possessed 2 binary features (*b*) that was coded as “1” and “0” when they were detected or not, respectively, to determine correlations between the presence of the microbe and the market-weight. The binary model was described as *y* = *β*_1_ *b* + *e* where **y,* β*_1_, *b* and *e* represented the market-weight per individual, the estimated effect for the binary effect, a binary feature and the residuals, respectively. Secondly, the correlation between OTU abundance and market-weight were analyzed using the quantitative model but only the subjects that contained the cecal microbiota associated with market-weight identified in the binary model were used. The quantitative model was expressed as *y* = *β*_2_ *q* + *e*, where *q* represented microbial abundance which are usually transformed into log_10_, *β*_2_ represented the estimated effect value of the quantitative model and *e* represented the residual. Combined with the effects of the binary model and the quantitative model, a meta-*p-*value was calculated using an unweighted Z-method. According to the minimum *p-*value for the binary model, quantitative model and meta-analysis, the corresponding allocation coefficient and final *p-*value was obtained. The Z-value was obtained from the Z distribution coefficient. Z > 0 represented a positive association between microbe and phenotype value and Z < 0 represented a negative association.

#### Other statistical analyses

We normalized the metabolite dataset though log_10_ transformation of the m/z values and then the module was constructed using a soft threshold Pearson correlation analysis (Langfelder and Horvath, 2008). This combined a topological overlap distance metric and average hierarchical clustering using weighted correlation network analysis (WGCNA) in the R package. The Matthews Correlation Coefficient (MCC) method of cytoHubba plug-ins in Cytoscape was used to identify the hub bacteria in the cecal microbiota network (Chin et al., 2014). LDA Effect Size (LEfSe) analysis^2^ was conducted to identify the cecal microbiota and serum metabolites that showed significant differences between high-and low-market-weight chicken groups (*p <* 0.05 and *LDA* > 2.0). All *p-*values of the multiple tests involved in this study were corrected by the Benjamini–Hochberg method. A total of 100 cross-validation tests were performed as previously described (Fu et al., 2015) to assess the proportion of cecal microbiota explaining the individual variation of market-weight. A random forest model (Ntree = 1,000) was employed to determine which OTUs could be used as microbial markers to distinguish high-and low-market-weight chickens (Guo et al., 2020). Spearman correlation analysis was used to identify relationships between cecal microbiota and serum metabolites or inflammatory cytokines.

## Results

### Experimental cohort (Qiandongnan Xiaoxiang chickens)

#### Differences in cecal microbiota diversity between high- and low-market-weight chickens

**Figure 1:**
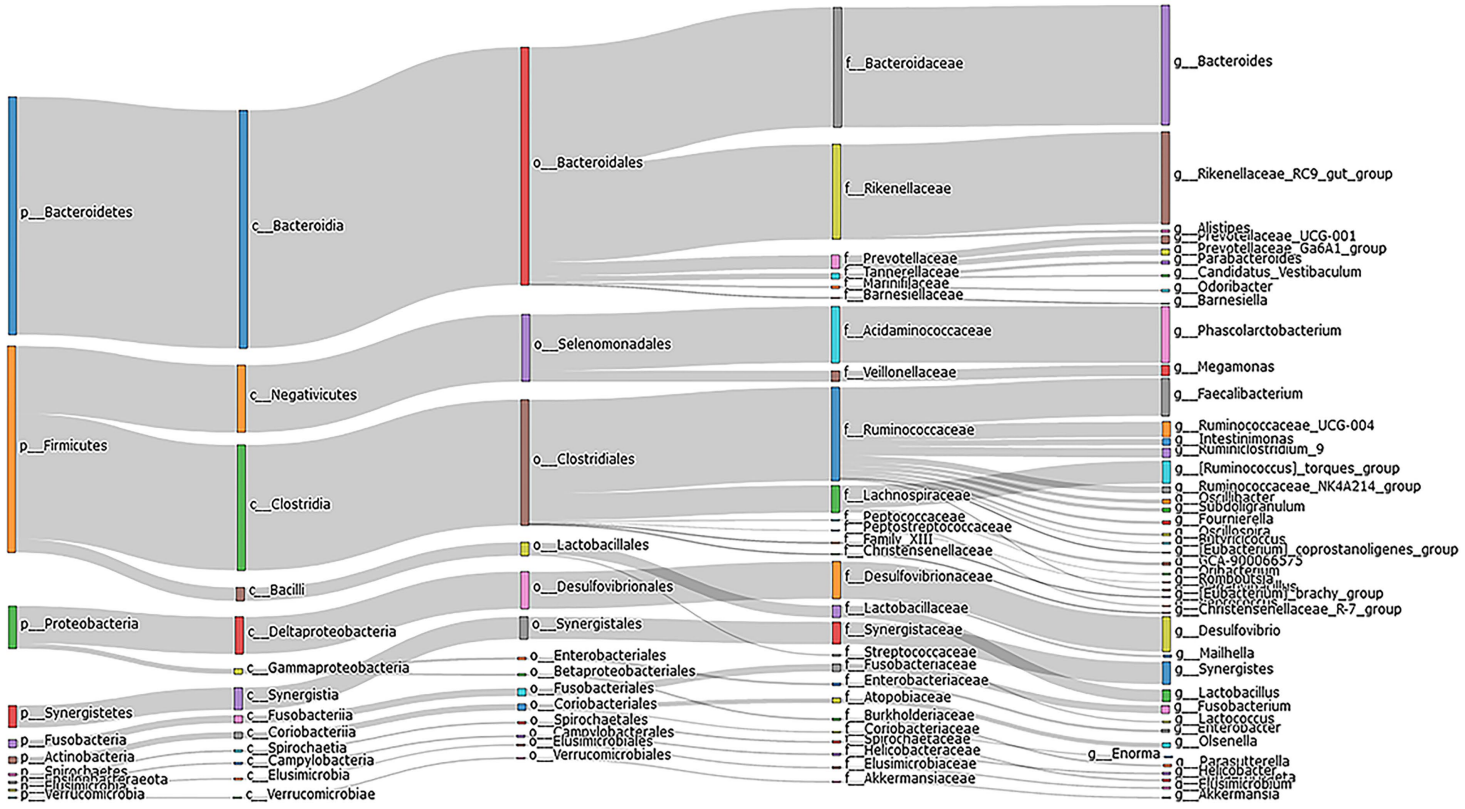
Sankey diagram depicting the bacterial composition of cecal content samples from Qiandongnan Xiaoxiang Chickens at 160 ± 3  days of age (*n* = 24). The colored columns from left to right represent the proportions of bacterial taxa from phylum to genus level.

**Figure 2:**
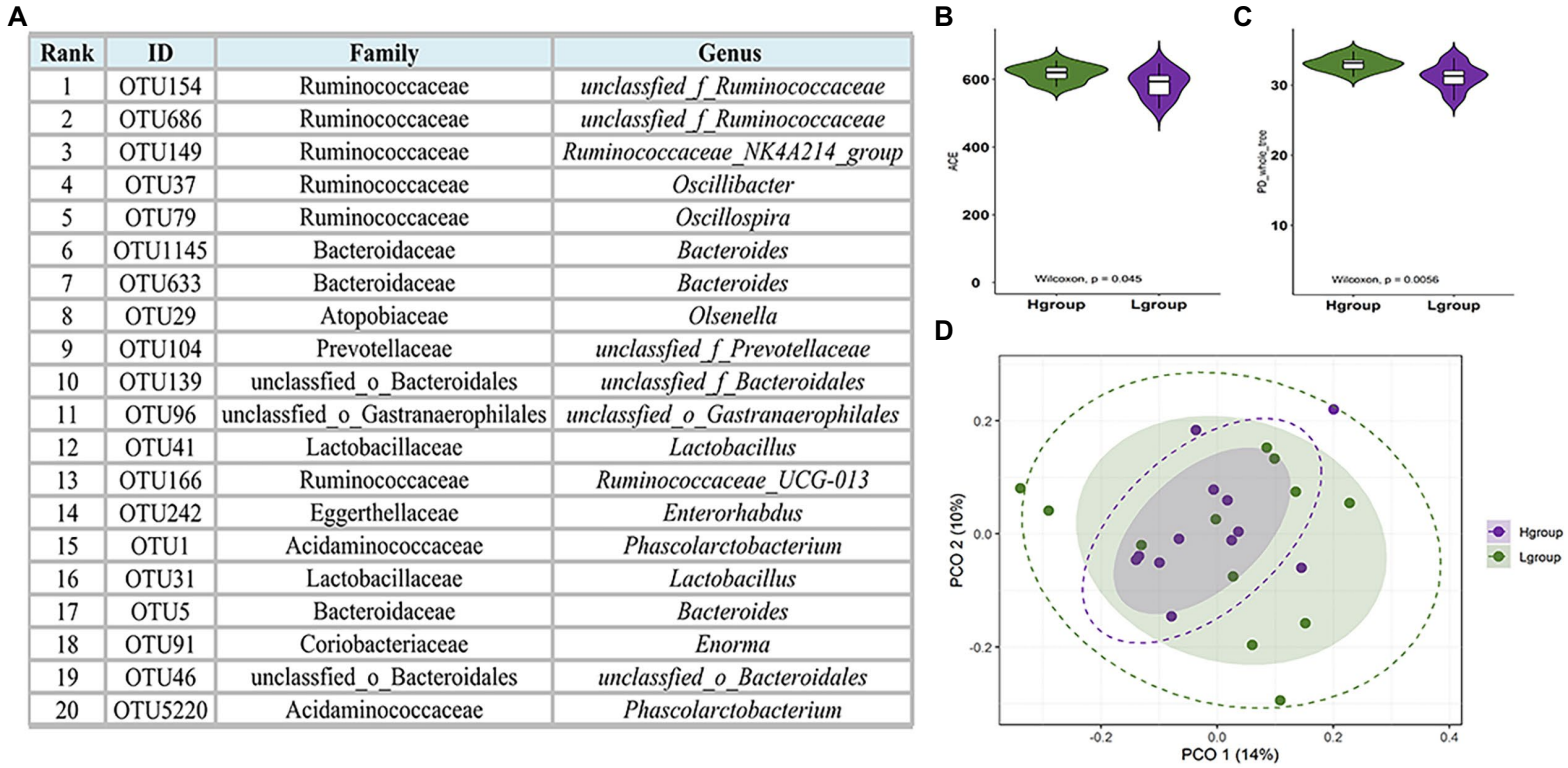
Hub cecal microbiota of Qiandongnan Xiaoxiang chickens at 160 ± 3  days of age and the difference of *α*-and *β*-diversity between chickens in high- (H group, *n =* 12) and low-market-weight (L group, *n =* 12) groups. **(A)** The 20 most abundant OTUs in the cecal microbiota network ranked using the Matthews Correlation Coefficient (MCC) method. Comparisons of **(B)** ACE and **(C)** PD indices between high-and low-market-weight chickens. **(D)** Principal coordinates analysis (PCoA) of microbial communities in cecal content samples based on unweighted UniFrac distances between high-and low-market-weight chickens.

An analysis of 24 cecal content samples of chickens from the experimental cohort generated 1,800,168 high-quality reads (75,007 reads per sample) and included 727 OTUs that were clustered according to 97% sequence identity. These OTUs were annotated to 16 phyla and 12 were present in all samples and 6 were present at a relative abundance >1%. The latter included Bacteroidetes (48.4%), Firmicutes (36.9%), Proteobacteria (5.8%), Synergistetes (2.6%), Spirochaetes (1.0%) and Actinobacteria (1.6%; Figure 1). The 5 most important OTUs for the hub microbiota of the Qiandongnan Xiaoxiang chickens were all members of the *Ruminococcaceae* family (Figure 2A). We then compared the *α-and β-*diversity of cecal microbiota between chickens with high-and low-market-weights. The *α-*diversity analysis indicated that scores for the ACE (Wilcoxon rank sum test, *p =* 0.03), Shannon (*p =* 0.01) and PD (*p =* 0.005) indices in the high-market-weight chickens were significantly greater than those of low-market-weight chickens (Figures 2B,C; Supplementary Figures S1A–C). In contrast, *β*-diversity comparisons using PCoA indicated no significant differences between high-and low-market-weight chickens (Figure 2D).

**Figure 3:**
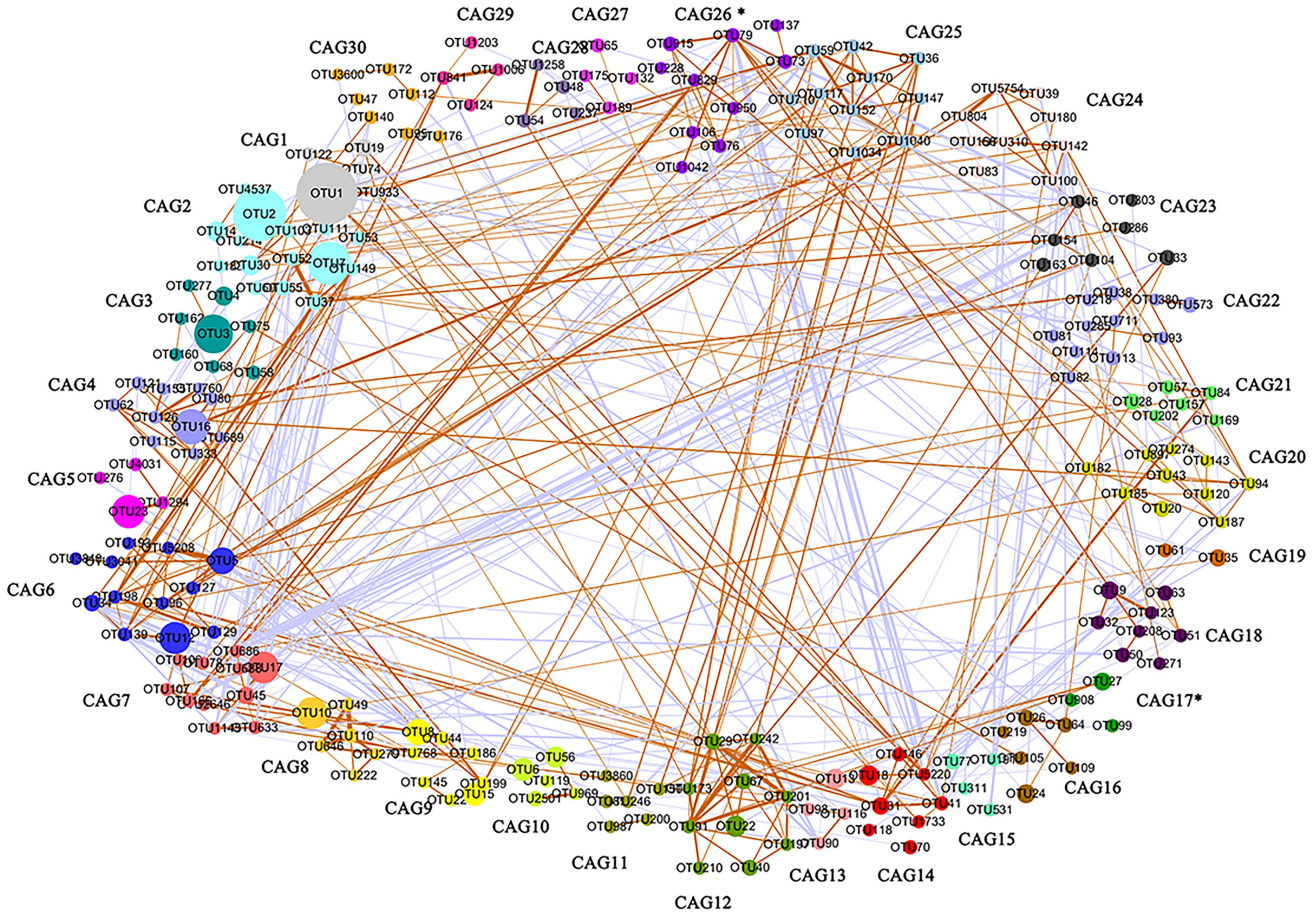
Co-abundance groups (CAG) in cecal microbiota based on the relative OTU abundance in Qiandongnan Xiaoxiang chickens at 160 ± 3  days of age (*n* = 24). A total of 261 OTUs were clustered into 30 CAGs using permutational multivariate analysis of variance. Asterisks (^*^) represent significant differences of relative OTU abundance in CAGs between high-and low-market-weight chickens. Node sizes are proportional to the average abundance of each OTU. The lines connecting two nodes represent SparCC correlations between the connected nodes with the line width representing the correlation magnitude. Brown and purple lines represent significantly positive and negative correlations between two OTUs, respectively, using an absolute value of correlation coefficient  > 0.55. Unconnected nodes were omitted.

One of our goals was the identification of cecal microbiota clusters that were related to market-weight. The gut microbiota is a huge and complex micro-ecosystem and the microbiota can directly or indirectly affect host physiological functions in the form of co-abundance groups (CAGs; Angulo et al., 2019). We therefore clustered the OTUs into CAGs based on their interaction network and obtained 261 OTUs after filtering that were clustered into 30 CAGs (Figure 3). We compared the average relative abundance of each CAG between high-and low-market-weight chickens and found that only the abundance of CAG17 (*p =* 0.04) was significantly different between the two groups and it was enriched in high-market-weight chickens. There were 5 OTUs in CAG17 and all belonged to the Clostridiales order (Supplementary Tables S2, S3). In particular, OTU234, OTU159 and OTU99 were annotated to *Eubacterium*, *Subdoligranulum*, and *Peptococcus*, respectively while OTU27 and OTU908 could not be annotated at the genus level. Significantly, differences in the average relative abundance of CAG26 between high-and low-market-weight chickens was near the level of significance (*p* = 0.08). CAG26 was a *Ruminococcaceae*-dominated CAG in which 8 / 11 OTUs belonged in this family that was enriched in high-market-weight chickens. OTU73, OTU106, OTU228, OTU829, OTU1042, and OTU79 were annotated to *Ruminococcaceae* UCG-005, *Ruminococcaceae* UCG-010, *Ruminococcaceae* UCG-014, *Ruminiclostridium*, *Negativibacillus* and *Oscillospira*, respectively. OTU950 and OTU136 were not annotated to specific bacteria at the genus level. These data suggested a beneficial effect of *Ruminococcaceae* on growth performance in chickens *via* the interaction network. The remaining 3 OTUs in CAG26 were OTU137, OTU76 and OTU1666 that were annotated to *CHKCI001* in Lachnospiraceae, Christensenellaceae and WCHB1-41, respectively.

#### Bacterial species with differential abundance between high- and low-market-weight chickens

**Figure 4:**
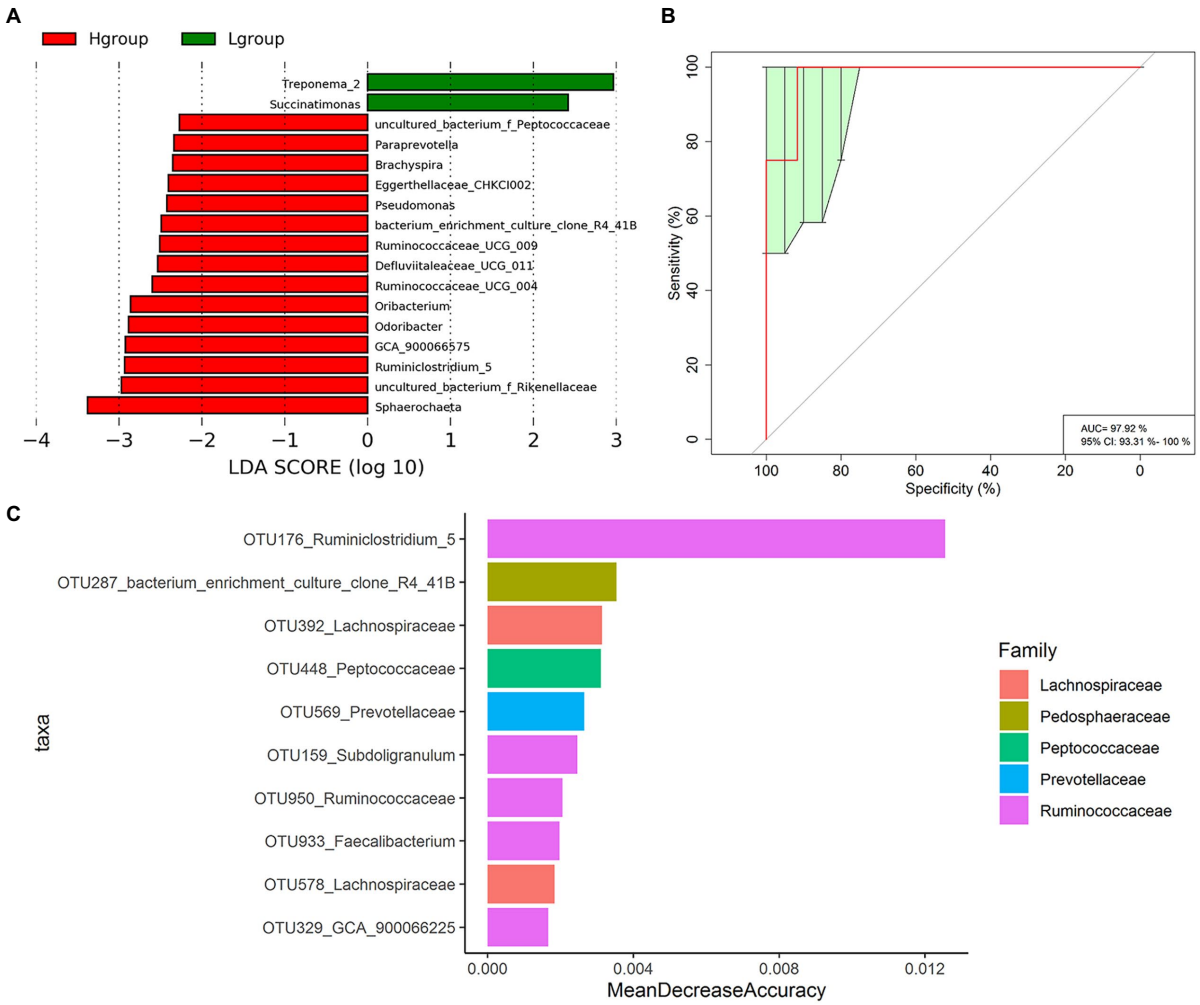
Specific OTUs of cecal microbiota related to market-weights of Qiandongnan Xiaoxiang chickens at 160 ± 3  days of age (*n* = 24). **(A)** LEfSe analysis of cecal microbiota members associated with H group and L group chickens at the genus level. **(B)** Receiver operating curves (ROC) for H and L group chickens. AUC = 97.92, 95% CI = 93.31%–100%. **(C)** The 10 most abundant OTU biomarkers that could discriminate H and L group chickens identified using the Random Forest model. Biomarker OTUs were ranked in descending order of importance relative to the model accuracy.

We further identified cecal microbiota members that were enriched in high-or low-market-weight chickens at the genus level. LEfSe analysis indicated that 17 genera possessed significant differences between the two groups. In particular, *Treponema_2* and *Succinatimonas* were enriched in the low-market-weight chickens while *Ruminococcaceae UCG-009, Ruminococcaceae UCG-004* and *Ruminiclostridium 5* were at high abundance levels for the high-market-weight chickens and consistent with the CAG results above. The high-market-weight group also contained *Oribacterium*, *GCA-900066575 Defluviitaleaceae UCG-011, uncultured_bacterium f Peptococcaceae* (order Clostridiales) and *Odoribacter, Paraprevotella* and *uncultured bacterium f Rikenellaceae* (order Bacteroidales). The other 5 genera enriched in the high-market-weight chickens were represented by the orders *Brachyspira, Pseudomonas, CHKCI002* and *bacterium enrichment culture clone R4-41B* and *Sphaerochaeta* (Figure 4A).

We applied a random forest classification analysis to identify the biomarker OTUs that might accurately distinguish the high-and low-market-weight chickens. We found that the 10 most prevalent OTUs could distinguish high-market-weight chickens from low-market-weight chickens with an accuracy of 97.92% (AUC value; Figures 4B,C). This group of 10 included 5 OTUs annotated to the *Ruminococcaceae* (OTU176, OTU159, OTU950, OTU933, and OTU329) annotated to *Ruminiclostridium 5*, *Subdoligranulum*, *Ruminococcaceae*, *Faecalibacterium* and *GCA-900066225*, respectively. It is noteworthy that OTU176 (*Ruminiclostridium 5*) possessed the highest score as a marker and this OTU also significantly differed between high-and low-market-weight chickens in the LEfSe analysis (see above).

#### Differential metabolite profiles between high- and low-market-weight chickens

**Figure 5:**
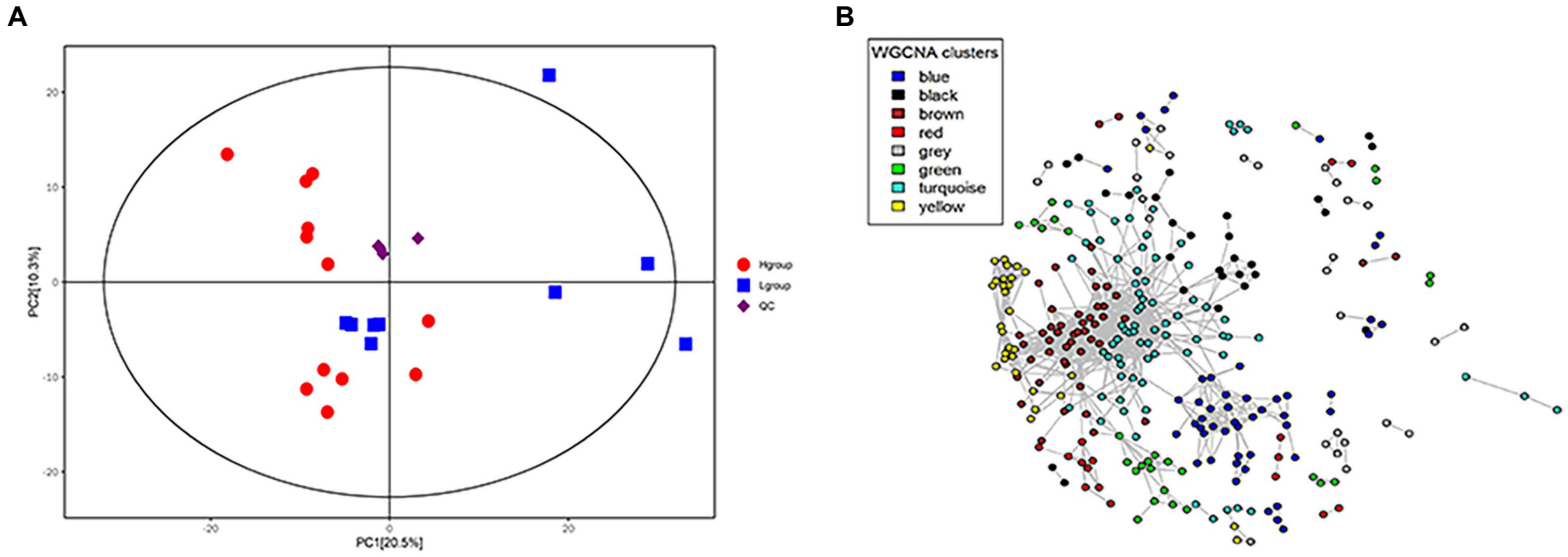
Differentiation of host serum metabolite profiles between H-group (*n* = 12) and L-group (*n* = 9) Qiandongnan Xiaoxiang chickens at 160 ± 3  days of age. **(A)** PCA plot of serum metabolite profiles **(B)** Co-occurrence network of serum metabolite features. The metabolites (nodes) are colored according to WGCNA module colors. Only those correlations with |*r*| > 0.2 between two edges are presented.

A total of 6,944 endogenous metabolites were obtained under ESI-and ESI^+^ modes and following relative standard deviation de-noising and annotation, 378 metabolites remained. PCA analysis indicated significant differences in the global metabolome between high-and low-market-weight chickens (Figure 5A). Specifically, 58 metabolite features were identified as market-weight-related (*p <* 0.001*, FDR <* 0.2) and 35 metabolites were enriched in serum samples of high-market-weight chickens while 23 metabolites were enriched in low-market-weight chickens (Supplementary Table S4).

**Figure 6:**
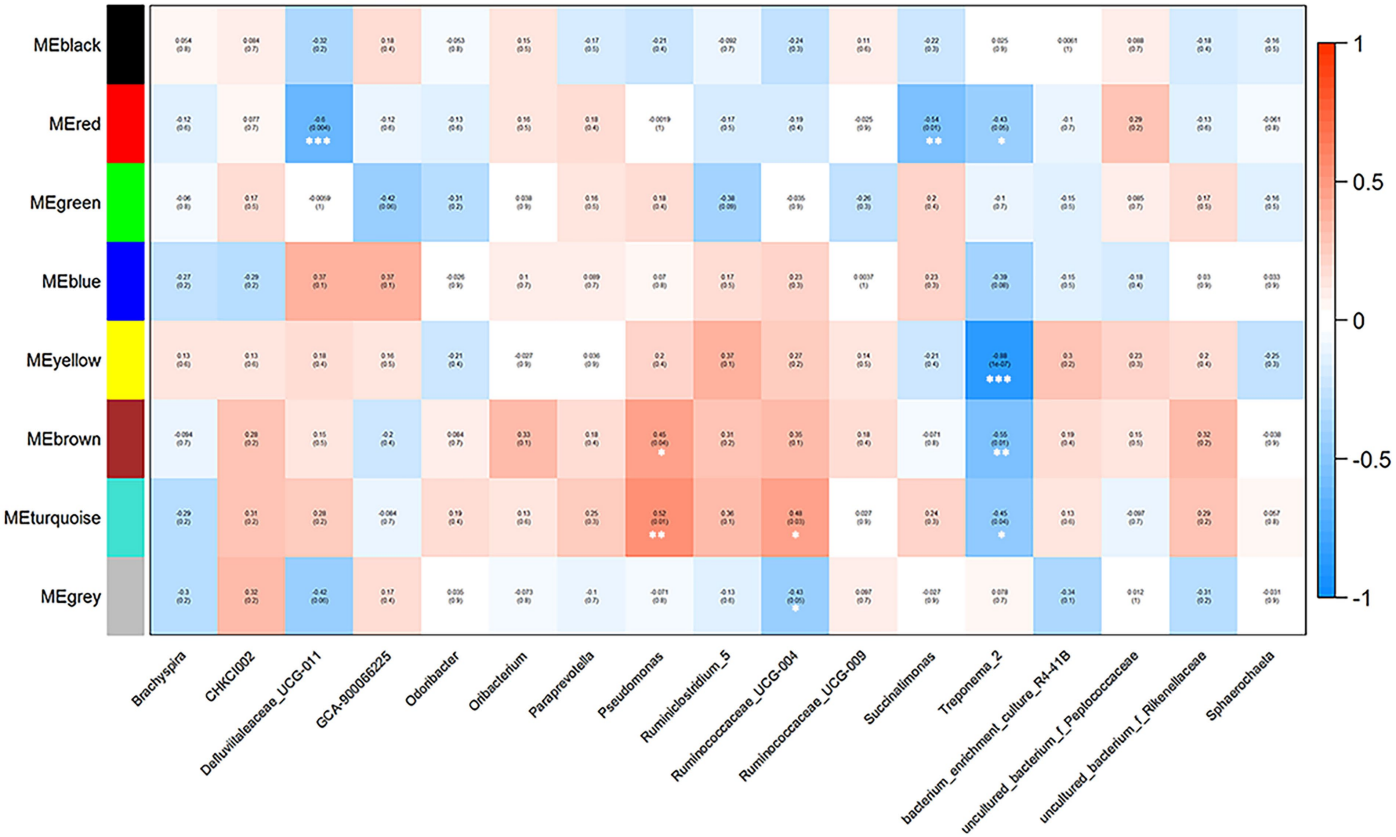
Correlations between metabolite modules and weight-related bacteria in Qiandongnan Xiaoxiang chickens at 160 ± 3  days of age (*n* = 21). Each box of the matrix indicates the correlation between one metabolite module and a weight-related bacterial taxon at the genus level. The correlation coefficients (*r*) and *p*-value are listed in the small boxes (*p < 0.05, **p < 0.01, ***p < 0.001). The color gradient represents the values of correlation coefficients (red for positive and blue for negative correlations).

Correlations between market-weight-related bacteria and host serum metabolites indicated that in the high-market-weight group, the enriched metabolites were dominated by nucleotide metabolites including primary and secondary bile acid metabolites [clustered in the turquoise (17/35 metabolites) and brown modules]. We also identified several significant correlations between metabolite modules and market-weight-related bacteria at the genus level. The turquoise module was found to be positively correlated with *Pseudomonas* (*p =* 0.01) and *Ruminococcaceae UCG-004* (*p =* 0.03) while negatively correlated with *Treponema 2* (*p =* 0.04). The brown module comprising primary and secondary bile acid metabolites was positively correlated with *Pseudomonas* (*p =* 0.04) but negatively correlated with *Treponema 2* (*p =* 0.01). Low-market-weight related metabolites were primarily clustered in the blue and gray modules. No differential bacteria were related with blue module of metabolites while the gray module was significantly negatively correlated with *Ruminococcaceae UCG-004* (*p =* 0.05; Figures 5B, 6).

#### Correlations between differential metabolites and differential cecal microbiota

**Figure 7:**
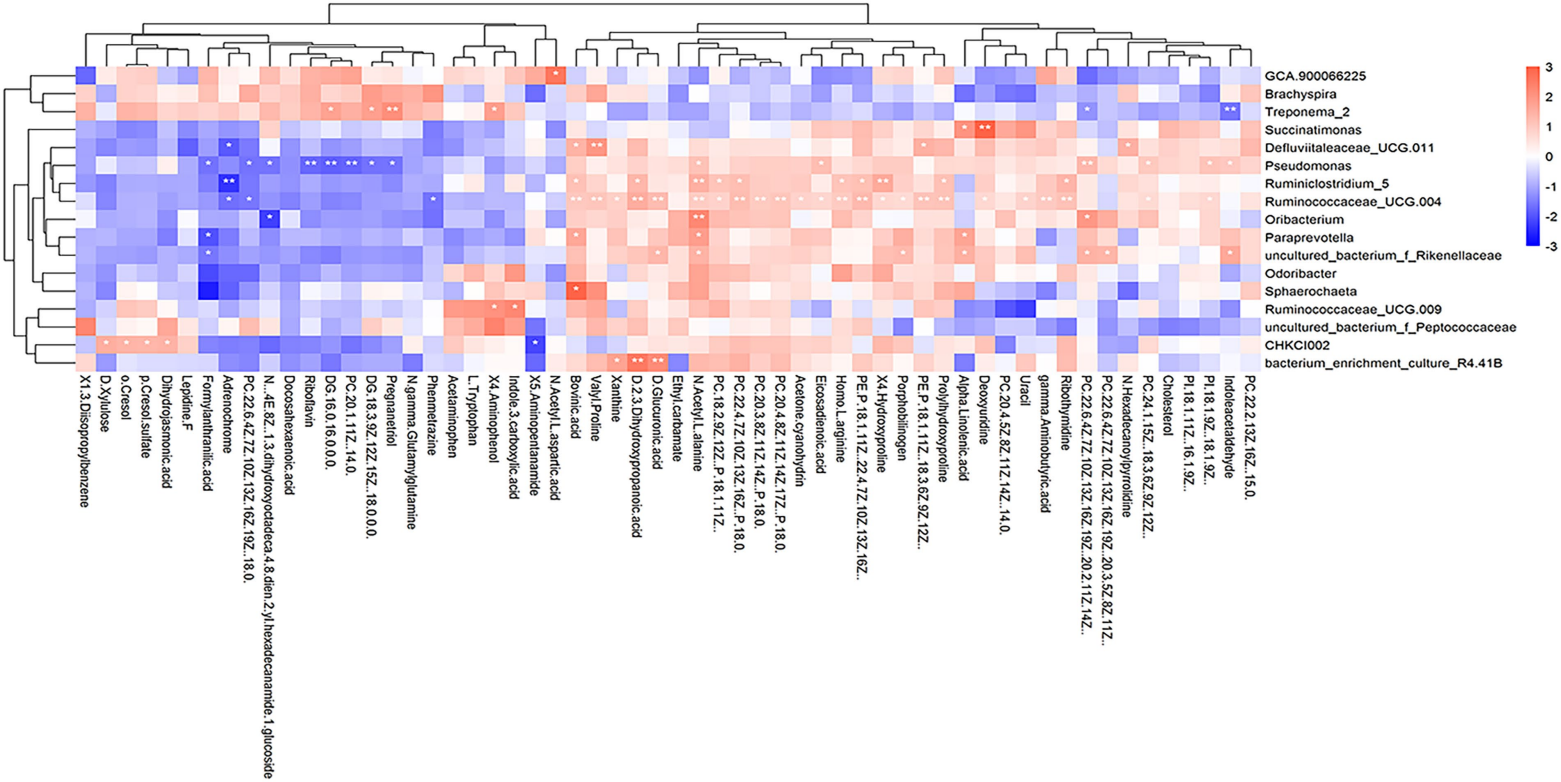
Heatmap depicting correlations between differential serum metabolites and differential species of cecal microbiota in Qiandongnan Xiaoxiang Chickens at 160 ± 3  days of age (*n* = 21). *p < 0.05, **p < 0.01, ***p < 0.001 were calculated using the Spearman’s rank correlation test.

We further explored whether the differential cecal microbiota possessed different metabolite profiles between high-and low-market-weight chickens. The results of differentiation analysis of serum metabolites showed that 58 differential metabolites were determined. And the results of a correlation analysis based on Spearman coefficients employed for 58 different metabolites and 17 different cecal microbiota showed that the genus *Ruminiclostridium 5* was significantly positively correlated with 10 metabolites including the amino acids prolylhydroxyproline, 4-hydroxyproline, homo-L-arginine as well as lipids but significantly negatively correlated with adrenochrome. *Ruminococcaceae UCG-011* was significantly correlated with 27 metabolites and might be one of the reasons the *Ruminococcaceae* were the hub cecal microbiota of Qiandongnan Xiaoxiang chickens. Interestingly, we found that *Treponema 2* was significantly enriched in the low-market-weight chickens and was also significantly positively correlated with the sex hormone pregnanetriol (Figure 7; Supplementary Table S5).

### Validation cohort (Guizhou yellow and Wumeng black-bone chickens)

#### Validating correlations of cecal microbiota and market-weight

**Figure 8:**
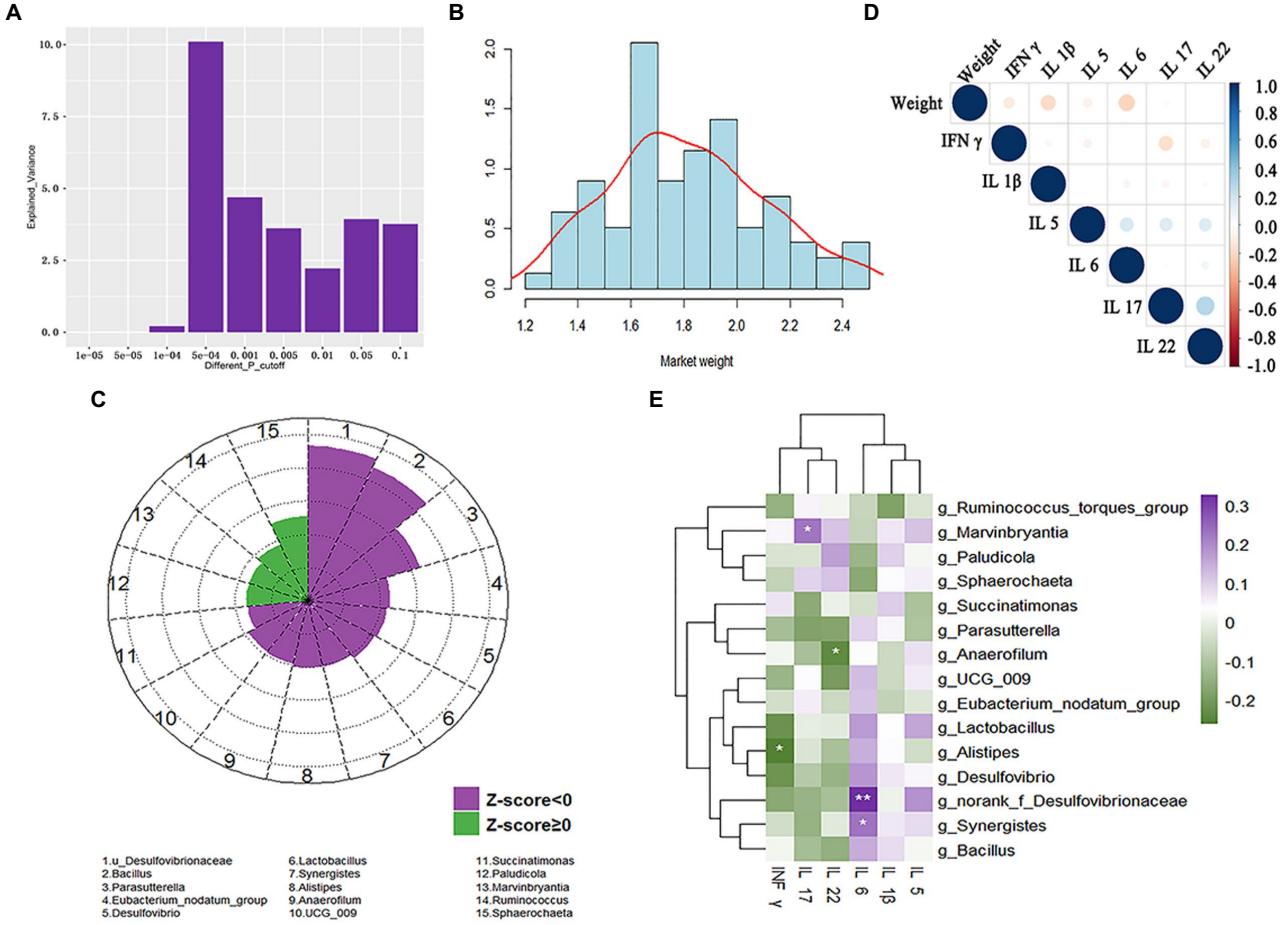
Correlation of market-weight (body-weight at 160 ± 3  days of age) with cecal microbiota and serum inflammatory cytokines in the validation cohort composed of 24 Wumeng black-bone chickens and 55 Guizhou yellow chickens. **(A)** Variation of market-weight explained by cecal microbiota under different significant thresholds. **(B)** Inter-individual variation in market-weight. **(C)** Cecal microbiota significantly associated with chicken market-weight. Associations between cecal microbiota and market-weight are shown as Z scores. Z < 0 and Z ≥ 0 indicates a negative association and a positive association, respectively. **(D)** Correlation between market-weight and 6 inflammatory cytokines. **(E)** Correlations between the abundance of cecal microbiota and host inflammatory cytokines quantified by Spearman correlation with Benjamini–Hochberg corrections. * *p <* 0.05, ** *p <* 0.01, *** *p <* 0.005.

We further verified associations between cecal microbiota and market-weight using the validation cohort of 55 Guizhou yellow and 24 Wumeng black-bone chickens (Supplementary Table S6). The cecal microbiota accounted for 10.1% of the variation among individuals with different market-weights at threshold of 5 × 10^−4^ (Figure 8A-B; Supplementary Table S7). We then corrected for two influencing factors (breed and sex) and a two-part model was used to identify the cecal microbiota associated with market-weight. We found 15 bacterial taxa were significantly correlated with market-weight and *Sphaerochaeta* (*LDA* = 2.53*, p =* 0.006), *Ruminococcus* (*LDA* = 1.86, *p =* 0.031)*, Marvinbryantia* (*LDA* = 1.70*, p =* 0.044) and *Paludicola* (*LDA* = 1.69, *p =* 0.045) were significantly and positively correlated (Supplementary Table S8). *Sphaerochaeta* was also enriched in the cecal microbiota of the high-market-weight chickens in the experimental cohort and was significantly associated with the conjugated linoleic acid, bovinic acid. Consistent results were obtained for *Succinatimonas* in both experimental cohort and validation cohort. *Succinatimonas* was enriched in the cecal microbiota of low-market-weight chickens and was significantly positively correlated with deoxyuridine (*r =* 0.63, *p =* 0.002) and α-linolenic acid (*r =* 0.43, *p =* 0.049). Additionally, 10 microbiotas including *norank_f_Desulfovibrionaceae* (*LDA* = −4.67, *p <* 0.001), *Parasutterella* (*LDA* = −3.25, *p <* 0.001) and *Eubacterium nodatum group* (*LDA* = −2.27, *p =* 0.012) were significantly negatively correlated with market-weight in the validation cohort (Figure 8C).

#### Relationships between inflammatory cytokines and market-weight

We also examined whether market-weight was affected by the presence of the inflammatory cytokines INF-*γ*, IL-1*β*, IL-5, IL-6, IL-17, and IL-22 (Broom and Kogut, 2018; Sun et al., 2018). Correlation analysis indicated that except for IL-22 (correlation coefficient 0.052), the other 5 cytokines were negatively correlated with market-weight and only IL-6 was significant with correlation coefficients of −0.235 (*p = 0.037*). The cecal microbiota *norank_f_Desulfovibrionaceae* (*r =* 0.33, *p =* 0.003) and *Synergistes* (*r =* 0.23, *p =* 0.04) that were negatively correlated with market-weight were extremely significantly correlated with IL-6 (Figure 8D; Supplementary Table S9).

## Discussion

The prohibition of antibiotics as growth promotors in food animals provides the impetus to develop alternative products to control gut bacterial pathogens. The gut microbiota is a key driver of growth performance in agricultural animals so these microorganisms and their metabolites are important candidate sources of probiotics and prebiotics (Ramayo-Caldas et al., 2016; Myer et al., 2017). Their use as growth promoters do not contribute to the development and spread of antibiotic resistance (Ma et al., 2022). In the current study, our experimental cohort (60 Qiandongnan Xiaoxiang chickens) and validation cohorts (24 Wumeng black-bone and 55 Guizhou yellow chickens) were established to profile phenotypic characteristics of the cecal microbiota and link these with market-weight. These indigenous breeds are important food sources in Guizhou, China and possess distinct phenotypic characteristics. Our goal was to exploit these differences and identify common beneficial bacteria that would be probiotic candidates.

The Qiandongnan Xiaoxiang chicken was selected for its superior meat flavor, small size and strong adaptability. The Guizhou Yellow broiler is a Chinese hybrid line (Weining ♀ × New Hampshire × Plymouth Rock ♂) that possesses high market weights and tender meat while the Wumeng black bone chicken is a meat and medicinal chicken breed that is listed in the *Poultry Genetic Resources in China* and possesses black tissues and bones. These indigenous chickens are popular with Guizhou consumers so they were chosen as the research objects in this study.

We found that *Bacteroidetes, Firmicutes, Proteobacteria, Synergistetes*, and *Spirochaetes* were the predominant cecal microbiota of Qiandongnan Xiaoxiang chickens. These results were similar to findings using the commercial breed Guangdong Yellow Broiler chickens except *Actinobacteria* replaced *Synergistetes* (Wen et al., 2019). The five most important OTUs of hub cecal microbiota in Qiandongnan Xiaoxiang chickens were annotated to the *Ruminococcaceae* and implicates this family as a core member of the cecal microbiota. *Ruminococcaceae* abundance was also significantly greater in high-market-weight chickens and this data combined with the CAG results linked this family to enhanced growth performance. *Ruminococcaceae* is one of the earliest described bacteria of the bovine rumen and are highly efficient carbohydrate decomposers and central to the degradation of resistant starch *via* fermentation to glucose and xylose (Pal et al., 2021). We also found that Rumi*nococcaceae* UCG-004 was significantly correlated with 27/58 differential metabolites and 10 of these were fatty acid metabolites (listed in Figure 7 with the prefix PC). Therefore, *Ruminococcaceae* in the cecum might also perform other physiological functions in the basic life activities of indigenous chickens. This family is one of the few known microorganisms that can transform primary bile acids into secondary bile acids (Jiang et al., 2022) and depletion of the *Ruminococcaceae* in human intestinal tracts is a marker for ulcerative colitis (Sinha et al., 2020). Together, these data implicate the *Ruminococcaceae* in intestinal health maintenance *via* alleviating intestinal inflammation through metabolism of specific fatty acids. These metabolic traits would specifically promote growth performance.

In both experimental and validation cohorts, we found a positive correlation between *Sphaerochaeta* and market weight. LEfSe analysis indicated that *Sphaerochaeta* was enriched in high-market-weight chickens in the experimental cohort confirming a key role in growth promotion. *Sphaerochaeta* is involved in glucose metabolism *via* glycolysis and the pentose phosphate pathway (Caro-Quintero et al., 2012) and was significantly positively associated with bovinic acid (a polyunsaturated conjugated linoleic acid; CLA isomer; Wang et al., 2019). CLA can be absorbed and rapidly deposited onto lipids and phospholipids in membranes (Kramer et al., 1998) and it possesses numerous biological functions including immunity enhancement in agricultural animals (Kuhnt et al., 2016). These beneficial effects link this genus to chicken growth performance *via* bovinic acid production.

In contrast to positive effects described above, the presence of *Treponema* and *Succinatimonas* in the cecum inhibited growth of Qiandongnan Xiaoxiang chickens. *Treponema* was previously implicated in altering the average daily gain of piglets in contrast to colonization by *Prevotella* and *Mitsuokella* (Ramayo-Caldas et al., 2016). A study of the Hadza people, a primitive hunting tribe in Africa, demonstrated that *Treponema* were significant dietary fiber degraders and this phenotype displayed significant gender differences (Schnorr et al., 2014). Other independent studies on agricultural animals have found that *Treponema* is an important marker of sex differences and might be related to alterations in sex hormone levels (He et al., 2019; Wang et al., 2021). In this study, we verified that *Treponema* was significantly positively correlated with the sex hormone pregnanetriol, indicating that a lower market-weight might be related to sexual maturity of the chickens. In the validation cohorts we also identified *Succinatimonas* as another important genus that was detrimental to Qiandongnan Xiaoxiang chicken growth. This genus of short, Gram-negative bacilli is enriched in cattle fed with grain hay (Morotomi et al., 2010). *Succinatimonas* can utilize only a few sugars such as glucose, maltose, dextrin and starch but no other carbohydrates, and its metabolites are largely succinic acid and a small amount of acetic acid (Morotomi et al., 2010). Excess succinic acid can result in diarrhea and thus lead to reduced growth.

Our combined results using the validation cohort indicated that high levels of the inflammatory cytokine IL-6 as well as the presence of *Synergistes* and *norank_f_Desulfovibrionaceae* were detrimental to growth. IL-6 levels were significantly positively correlated with these two genera and suggest they promote inflammation and this could seriously reduce growth performance due to decreased feed intake and abnormal digestion and absorption (Jiang et al., 2010; Chen et al., 2021). Probiotics such as *Lactobacillus* can mitigate inflammation in mice by regulating cytokine secretion (Lamas et al., 2016). And gut microbiota had positive effects in the chicken caecum to promote growth by mitigating intestinal inflammatory (Ayalew et al., 2022). The genus *Synergistes* is widely distributed in the natural environment and are characterized by their ability to degrade amino acids and may perform this function in natural ecosystems (Godon et al., 2005). Additionally, *Synergistes* is a normal part of human and animal microflora and has been linked to mucosal infections (Kumar et al., 2003).

The second genus we linked to decreased growth was the sulfate-reducer *Desulfovibrio* that utilize sulfate as the terminal electron acceptor for ATP synthesis. H_2_S production by this genus has also been linked to increased inflammation of the intestinal epithelium in humans (Verstreken et al., 2012; Murros et al., 2021). Taken together, we identified specific cecal microbiotal members that affect the growth performance of indigenous chickens *via* inflammatory cytokine regulation.

## Conclusion

Our findings demonstrated that cecal colonization by *Sphaerochaeta* improved growth performance of Guizhou indigenous chicken by promoting bovinic acid metabolism. In contrast, colonization by *Synergistes* and *norank_f_Desulfovibrionaceae* in cecum reduced growth performance by inducing the production of the inflammatory cytokine IL-6. These results provide novel insights into the development of antibiotic alternatives to improve chicken growth performance and deepen our understanding of the physiology of native Guizhou chicken breeds.

## Data availability statement

All sequencing data has been uploaded to the Chinese gene pool database (China National GeneBank database, CNGBdb; https://db.cngb.org/cnsa/) with project number CNP0003145.

## Ethics statement

The animal study was reviewed and approved by Animal Care and Use Committee in Guizhou University.

## Author contributions

ZW and LW conceived and designed the experiments. LW, SL, ShenhY, and YY performed the experiments. ZW, FZ, HL, ShengY, and XC analyzed the data. ZW and LW wrote the paper. All authors contributed to the article and approved the submitted version.

## Funding

This work was Supported by the National Natural Science Foundation of China (32260829 and 32160853), Guizhou Provincial Science and Technology Project (QKH-ZK2022-113), the Guizhou University Cultivation Project (GZPY-2020-58), the Natural Science Research Project of Guizhou Provincial Department of Education (QJJ-ZK2022-061), Scientific Research Fund for Talents Recruiting of Guizhou University (202137), Natural Science Special (Special Post) Research Fund Project of Guizhou University (202221), and Guizhou Provincial Science and Technology Project (QKH-ZC2022-key34).

## Conflict of interest

The authors declare that the research was conducted in the absence of any commercial or financial relationships that could be construed as a potential conflict of interest.

## Publisher’s note

All claims expressed in this article are solely those of the authors and do not necessarily represent those of their affiliated organizations, or those of the publisher, the editors and the reviewers. Any product that may be evaluated in this article, or claim that may be made by its manufacturer, is not guaranteed or endorsed by the publisher.

## Footnotes

^1^ https://hmdb.ca/

^2^ http://huttenhower.sph.harvard.edu/galaxy
