# Supplementary figures and images for: Metabolic and inflammatory linkage of the chicken cecal microbiome to growth performance
Source: Front Microbiol. 2023 Feb 23;14:1060458. doi: 10.3389/fmicb.2023.1060458 (PMC9995838; doi:10.3389/fmicb.2023.1060458)

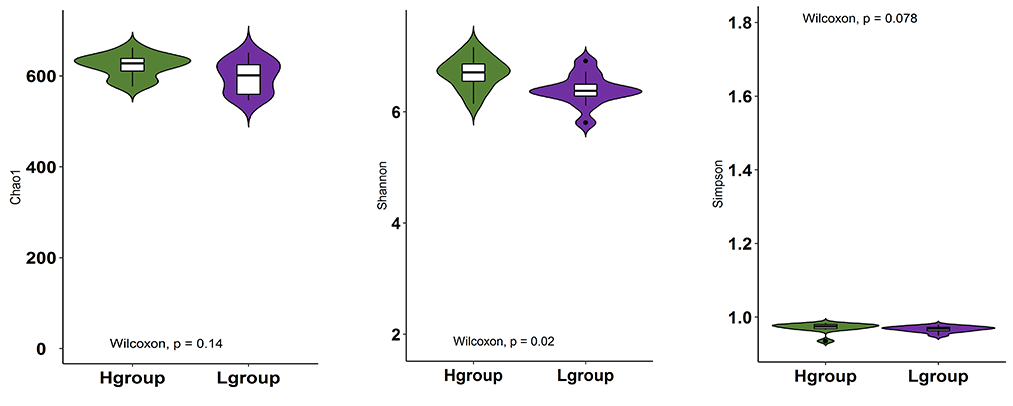

Supplement: Supplementary file 2 [file Image_1.TIF]
